# Perspectives on Physical Activity in Nephrology: An Explorative Cross‐Sectional Survey of Adults With Chronic Kidney Disease and Nephrology Nurses

**DOI:** 10.1111/jorc.70090

**Published:** 2026-11-05

**Authors:** Marie Skovgaard Jakobsen, Jeanette Finderup, Hannah ML Young, Mathias Pedersen, Stig Molsted

**Affiliations:** ^1^ Faculty of Health and Medical Sciences University of Copenhagen Copenhagen Denmark; ^2^ Department of Renal Medicine, Aarhus University Hospital, Denmark & Department of Clinical Medicine Aarhus University Aarhus Denmark; ^3^ NIHR Leicester Biomedical Research Centre University of Leicester and University Hospitals of Leicester NHS Trust Leicester UK; ^4^ Diabetes Research Centre, College of Life Sciences University of Leicester Leicester UK; ^5^ Therapy Department University Hospitals of Leicester NHS Trust Leicester UK; ^6^ Department of Clinical Research Copenhagen University Hospital–North Zealand Denmark; ^7^ Department of Physiotherapy and Occupational Therapy Copenhagen University Hospital–North Zealand Denmark; ^8^ Department of Clinical Medicine University of Copenhagen Copenhagen Denmark

**Keywords:** chronic kidney disease, haemodialysis, nurse, physical activity, physical function

## Abstract

**Background:**

Persons with chronic kidney disease are often physically inactive, despite evidence supporting the benefits of physical activity. However, implementation of physical activity in clinical practice remains limited.

**Objectives:**

To investigate and compare informational needs and priorities regarding physical activity among persons with chronic kidney disease and nephrology nurses.

**Methods:**

This cross‐sectional survey included adults with chronic kidney disease and nephrology nurses in Denmark. Participants reported their need for information on physical activity, preferred educational formats and preferred clinical monitoring approaches.

**Results:**

A total of 82 persons with chronic kidney disease and 107 nurses participated. Both groups expressed a need for more information on exercise modalities, safety, and the effects of physical activity. Nurses more frequently requested information on exercise modalities (78.5% vs. 63.4%, *p* = 0.013) and motivational strategies (79.4%). Patients more often preferred physical activity level as a clinical monitoring parameter (69.5%), whereas nurses preferred physical function (66.4%) (*p* = 0.013). Audiovisual materials were widely requested by both groups.

**Conclusions:**

Persons with chronic kidney disease and nephrology nurses share substantial informational needs regarding physical activity, although differences exist in preferred monitoring approaches. Developing aligned educational resources and incorporating motivational strategies may support implementation of physical activity in clinical practice. Due to exploratory nature, the findings of this study should be interpreted as hypothesis‐generating rather than confirmatory.

## 1 Introduction

Chronic kidney disease (CKD) is associated with physical inactivity, reduced health‐related quality of life (HRQoL), and impaired physical function (Lambert et al. 2022). It has been estimated that 66%–89% of the patients living with CKD report physical inactivity (Stevens et al. 2023). In patients receiving maintenance dialysis, low levels of physical activity (PA) including exercise training are associated with poorer HRQoL, lower physical function, more bodily pain, and increased hospitalization (Lambert et al. 2022). In patients living with CKD who are not undergoing dialysis, inactivity is associated with frailty and disability (Bellizzi and Regolisti 2022). There is good evidence of the beneficial effects of exercise training on aerobic capacity (Ferrari et al. 2023), physical function (Pu et al. 2019), and HRQoL (Hu et al. 2023) in patients receiving dialysis, and there are also positive effects in patients with transplantation (Zhang et al. 2023) and in patients without kidney replacement therapy Heiwe and Jacobson (2011). The exercise training can be provided with or without supervision and in or outside nephrology centres. One way to provide exercise training is with a home‐based nurse‐led intervention (Junqué‐Jiménez et al. 2023). However, implementation of exercise interventions in clinical nephrology is still challenged by different barriers.

### 1.1 Literature Review

The implementation challenges include barriers at patient, staff, and health‐care system levels (Young et al. 2025). On an organizational level, it has been reported that there are more PA barriers, that demands a focus in clinical practice (Young et al. 2025). Barriers such as health‐care professionals may not have sufficient knowledge of exercise prescriptions (type, intensity, duration), safety and contraindications with training, and behaviour‐change techniques including motivational interviewing. There may also be issues with funding as CKD does not have the same amount of attention as other chronic diseases, including heart disease, diabetes, chronic obstructive pulmonary disease, and cancer (Bennett et al. 2022). A recent study assessed that access to exercise training for persons with CKD remains suboptimal because of inadequate funding, limited rehabilitation workforce capacity, low awareness of available services, and a lack of policy‐driven integration of rehabilitation into routine nephrology care (Wong and McMahon 2026).

In the GoodRENal project it was found that persons with CKD wanted more knowledge regarding effects and safety of different exercise modalities, and the staff recommended that training support should be multidisciplinary and individualized (Mesa‐Gresa et al. 2024). A barrier to the multidisciplinary approach is, however, that physiotherapists are not a part of the treatment team in nephrology as reported in the EUSUREX study (Clyne et al. 2025). In a guideline, Baker and colleagues highlighted a persistent evidence‐to‐practice gap, whereby exercise is widely recommended in CKD guidelines but remains inadequately implemented in routine kidney care (Baker et al. 2022). A recent study provided recommendations about how to implement training to persons with CKD, and in the same evaluation more knowledge from implementation research within the field was wanted (Wilkinson et al. 2025).

In a previous study, nephrology nurses and nephrologists had positive attitudes to exercise training and PA (Molsted et al. 2023). Despite these positive attitudes, nurses had reported that they needed more tailored information about the effects of PA, exercise modalities, safety, and guidance on how to motivate patients to be active. By only including the views of clinical staff on PA, there is a knowledge gap in what the persons with CKD prefer and what they might need when it comes to exercise training and PA. Furthermore, clinical staff may also need guidance on how to assess PA in clinical practice in cooperation with persons living with CKD. Support to persons with CKD to become more physically active should be provided by more health‐care staff members, among them nurses, medical doctors, physiotherapists, and dieticians. However, in many nephrology centres, nurses are a crucial group, as they have very frequent contact to the persons with CKD, and as many centres may not have included physiotherapists in the contact to their patients as stated above.

The aim of this survey was to investigate what information persons with CKD and nephrology nurses need and prioritize regarding PA and exercise training in clinical practice. Furthermore, the aim was to compare the parameters of views and the need for information about PA between persons with CKD and nurses.

## 2 Methods

### 2.1 Study Design

This study was an exploratory cross‐sectional study with self‐reported outcomes from a survey. The study is reported in accordance with the Strengthening The Reporting of Observational Studies on Epidemiology statement (STROBE) guideline for reporting observational studies (Elm et al. 2007). Data from persons with CKD were collected between 4 February 2025, and 19 March 2025. Data from nurses were collected between 10 January 2025, and 21 February 2025.

### 2.2 Setting

The study was approved by the Data Protection Agency (record no. p‐2024‐17596), and it was conducted in accordance with rules of the Danish National Committee on Health Research Ethics (record no. F‐24066552). Data were collected and stored in a secure web‐based REDCap database designed to support data capture for research studies hosted in The Capital Region, Denmark (Harris et al. 2019).

### 2.3 Participants

All participants were invited to complete an electronic questionnaire via a QR code. The participants received written information about the study followed by access to the questionnaire.

Persons with CKD were invited to the study via email by the Danish Kidney Association. To increase the number of participants with CKD, a haemodialysis (HD) unit was approached, and its patients were invited to participate if they had not volunteered via the invitation from the Danish Kidney Association. Inclusion criteria for persons with CKD were a minimum age of 18 years and living with CKD and treated with maintenance dialysis (*Kidney Disease: Improving Global Outcomes* stage 5), transplantation, or eGFR < 30 mL/min/1.73 m^2^ without kidney replacement therapy (*Kidney Disease: Improving Global Outcomes* stage 4‐5). Exclusion criteria were inability to speak Danish, and mental health diseases that would make the participation difficult according to an assessment by the staff.

Nurses from Danish hospital departments of nephrology were recruited via the Danish Society of Nephrology Nurses (FS Nefro) after an introduction to the study at the society's annual meeting in November 2024. Members of the meeting were asked to invite nephrology nurses, who did not participate in the meeting, to complete the questionnaire. All employed nurses in Danish departments of nephrology were welcome to participate with no further inclusion or exclusion criteria.

### 2.4 Variables and Measurements

The survey consisted of 14 questions to persons with CKD and 13 questions to nurses.

In the survey, the participants were asked whether they wished to receive more information about (1) effects of exercise training and PA for persons with CKD, (2) exercise modalities for persons with CKD, and (3) safety in exercise training and PA for persons with CKD (reply options: yes; no; do not know), and (4) written or video material about exercise training and PA for persons with CKD and (5) whether they preferred self‐reported PA or physical function, or nurse‐reported frailty in a patient record registration related to exercise training and PA (reply options: yes; no; other). In addition, the nurses were asked whether they wished to receive more information about strategies to support patient motivation with exercise training and PA. In the questionnaire, it was possible to add multiple treatments, as persons living with CKD often have been through more treatment regimens. For nurses, it was possible to add multiple workplaces, because nurses often work with multiple groups of persons living with CKD. The participants' levels of PA were measured using a question from The Danish Health and Morbidity Survey (Jensen et al. 2019): the participants were asked about weekly leisure and transport time spend on moderate‐to‐vigorous PA defined as activities with increased breathing and exemplified as brisk walking, bicycling, heavy gardening, running, or recreational sports. The reply options were: < 30 min; 30–89 min; 90–149 min; 150–299 min; ≥ 300 min. In addition, the persons with CKD were asked two additional questions from the Danish Health and Morbidity Survey (Jensen et al. 2019): Do you want to be more physically active? (reply options: yes; no; do not know), and: How is your physical capacity? (reply options: Likert scale). An overview of the questions in the first part of the survey is presented in Table 1 and the full Danish questionnaire is available on request from the corresponding author.

**Table 1:** Items in questionnaire.

Item	Persons with CKD	Nephrology Nurses
Do you need more information about effects of exercise training and physical activitiy to persons with CKD	x	x
Do you need different possible exercise modalities to persons with CKD	x	x
Do you need more information about safety in exercise training and physical activity to persons with CKD	x	x
Do you need more information about exercise training and physical activity motivation to persons with CKD		x
Do you need more information about exercise training and physical activity to persons with CKD in written material	x	x
Do you need more information about exercise training and physical activity to persons with CKD in video materials for smartphone, tablet or PC?	x	x
Do you need more information about exercise training and physical activity to hand out to persons with CKD		x
If there should be a registration related to exercise training and physical activity in the patient record, would you suggest: − Physical activity estimated from a questionnaire filled out by patients. − Physical function estimated from a questionnaire filled out by the patients. − Frailty assessment estimated by a screening tool filled out by a nurse. − Other	x	x

### 2.5 Bias

The questionnaire was assessed for face validity by persons with CKD, and for content validity by nephrology nurses before being used. The face validity was assessed in five persons with CKD, who should represent that group and assess whether the tool could be understood, and they found the questions easy to understand. The content validity was assessed by five nephrology nurses, who should represent their profession and asses the tool as professions within their field, and they found the questions relevant to ask to persons with CKD and nephrology nurses. Thus, no changes to the developed questionnaire were made.

The questionnaire was not further psychometrically evaluated, for example, in a test–retest reliability and/or an internal consistency reliability test on greater number of participants than in the face and content validity assessments, as the study was explorative and primarily presented descriptive data.

The PA level was self‐reported, and this method may have introduced recall or measurement bias.

### 2.6 Study Size and Statistical Methods

The study was considered exploratory and performed without a sample size calculation. The data analyses included descriptive, comparative, and a regression analysis. A chi‐square test was used to compare the frequencies of the categorical outcomes' views and the need for information about PA between persons with CKD and nurses. Association between groups (persons with CKD or nurses) and level of reported moderate‐to‐vigorous PA minimum 150 min/week as recommended by the World Health Organization was analysed using a multiple logistic regression model adjusted for age and sex. The regression model was performed to compare the two groups' PA levels with the adjustments used to assess their relation to PA. Data were described in groups for those with CKD and nurses and presented as mean ± standard deviation (SD), median (inter quartile range (IQR)), *n* (%), and mean [CI 95%]. The data analysis was performed using Excel and SPSS. A significance level of *p* < 0.05 was considered significant. All *p*‐values were corrected for multiple comparisons using Benjamini‐Hochberg False Discovery Rate. However, given the exploratory nature of the study, *p*‐values are reported descriptively and should be interpreted with caution.

## 3 Results

### 3.1 Participants and Descriptive Data

82 persons with CKD and 107 nurses participated in the study. The participants' characteristics are presented in Table 2. The mean age of those with CKD was 57.3 ± 12.1 years and 62.5% identified as women (*n* = 51). The nurses were 47.4 ± 12.4 years old and 98.1% identified as women (*n* = 105). The persons with CKD could in the questionnaire report more treatment modalities: 52.4% (*n* = 43) were in active treatment with HD; 25.6% (*n* = 21) received peritoneal dialysis; 41.5% (*n* = 34) patients had a transplantation, and 25.6% (*n* = 21) received medical management of their CKD. The mean eGFR was 22.7 ± 23.3 ml/min/1.73 m^2^. 60.7% (*n* = 65) of the nurses had more than 10 years of experience in nephrology.

**Table 2:** Characteristics and descriptive parameters of participants included in the study.

Variables	Persons with CKD (*n* = 82)	Nephrology nurses (*n* = 107)
Age (years)	57.3 ± 12.1	47.4 ± 12.4
Sex (female)	51 (62.2%)	105 (98.1%)
Treatment modality		
Haemodialysis	43 (52.4%)	
Peritoneal dialysis	21 (25.6%)	
Transplantation	34 (41.5%)	
Medical management	21 (25.6%)	
Other	2 (2.4%)	
Time in treatment	8.4 ± 11.3	
eGFR (mL/min/1.73 m^2^)	22.7 ± 23.3	
Work experience in nephrology (years)		
0–2		15 (14.0%)
2–5		17 (15.9%)
5–10		10 (9.3%)
> 10		65 (60.7%)
Place of work		
Haemodialysis		83 (77.6%)
Peritoneal dialysis		11 (10.3%)
Transplantation		10 (9.3%)
Outpatient clinic		13 (12.1%)
Hospital unit		16 (15%)
Other		5 (4.7%)

*Note:* Data are presented as mean ± SD and *n* (%).

### 3.2 Outcome Data and Main Results

Figure 1 shows that 57.3% (*n* = 47) of the persons with CKD and 64.5% (*n* = 69) of the nurses desired more information about the effects of PA (*p* = 0.705). 63.4% (*n* = 52) of persons with CKD and 78.5% (*n* = 84) of the nurses desired information on exercise modalities (*p* = 0.013). 52.4% (*n* = 43) of persons with CKD and 57% (*n *= 61) of the nurses desired more information about safety in exercise training (*p* = 0.705). 79.4% (*n* = 85) of the nurses desired more information on how to motivate persons with CKD to exercise. 46.3% (*n* = 38) of persons with CKD and 66.4% (*n* = 71) of the nurses desired written material about exercise training in CKD (*p* = 0.013) (see Figure 2). 54.9% (*n* = 45) of persons with CKD and 69.2% (*n* = 74) of the nurses desired audiovisual material about exercise training in CKD (*p* = 0.079). 69.5% (*n* = 57) of persons with CKD and 49.5% of nurses suggested that the level of PA should be measured and monitored in clinical practice (*p* = 0.013) (Figure 3). 46.3% (*n* = 38) of persons with CKD and 66.4% (*n* = 71) of nurses suggested physical function should be measured and monitored (*p* = 0.013). 40.2% (*n* = 33) of persons with CKD and 39.3% (*n* = 42) of nurses suggested that frailty should be measured and monitored (*p* = 0.890). Data for PA are presented in Table 3. A minimum of 150 min of moderate‐to‐vigorous PA per week was reported by 19.5% (*n* = 16) of persons with CKD and 47.7% (*n* = 51) of nurses. 19.5% (*n* = 16) of persons with CKD reported that their physical capacity was good or very good. 75.6% (*n* = 62) of persons with CKD wished to be more physically active.

**Figure 1:**
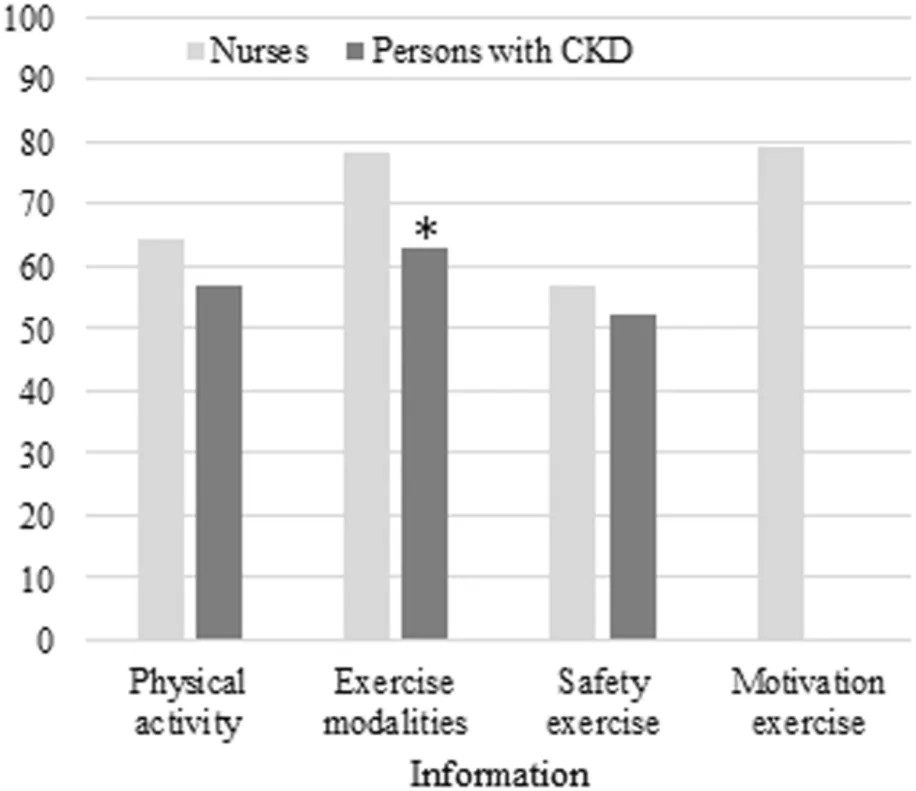
Persons with CKD and nephrology nurses wishing more information about the effect of physical activity, different exercise modalities, and safety in exercise training, and nurses wishing more information about how to motivate persons with CKD to be more physically active in percentages. *Difference between persons with CKD and nephrology nurses *p* < 0.05. CKD, chronic kidney disease.

**Figure 2:**
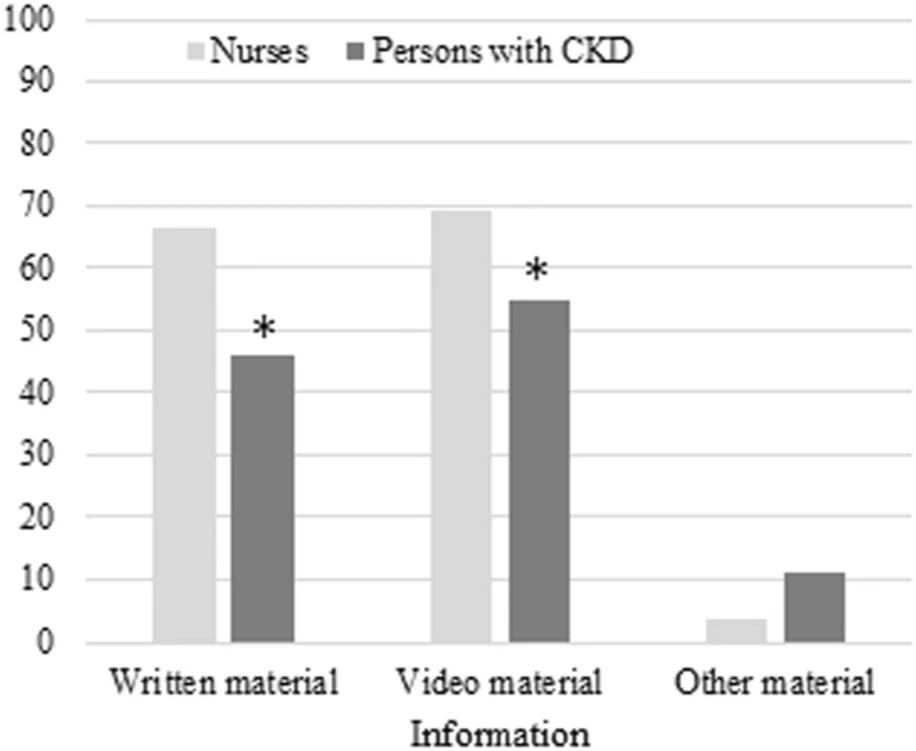
Persons with CKD and nephrology nurses' preferences of types of information material about physical activity and exercise training in percentages. *Difference between persons with CKD and nurses *p* < 0.05. CKD, chronic kidney disease.

**Figure 3:**
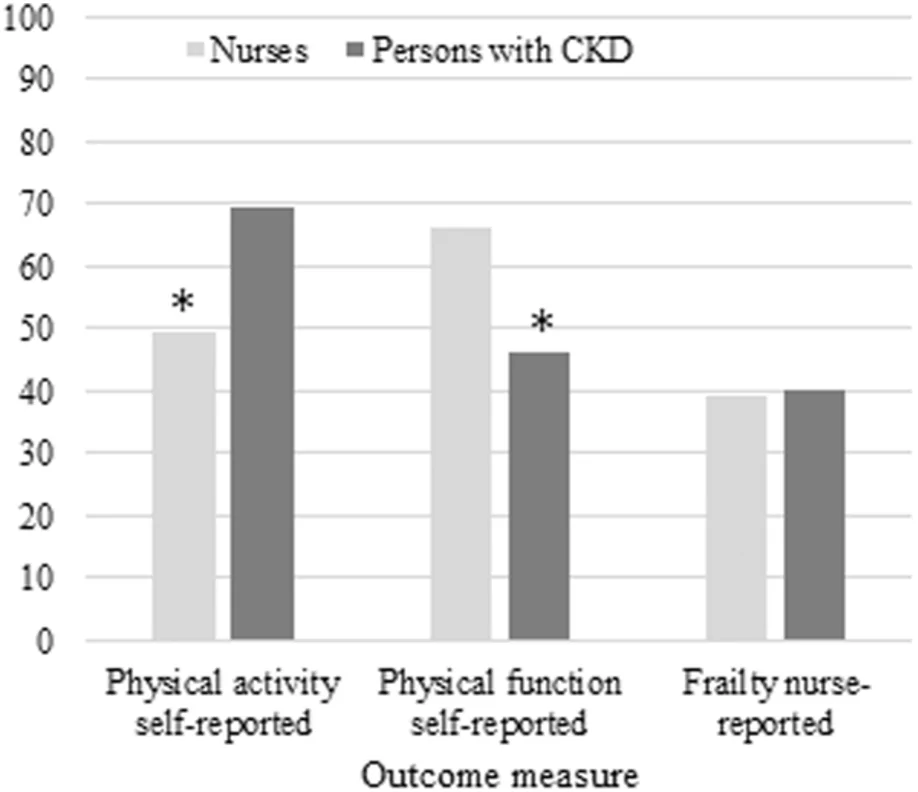
Persons with CKD and nephrology nurses' preferences for outcome measures in clinical practice in percentages. *Difference between persons with CKD and nephrology nurses *p* < 0.05. CKD, chronic kidney disease.

**Table 3:** Characteristics and descriptive parameters of participants included in the study.

Variables	Persons with CKD (*n* = 82)	Nephrology nurses (*n* = 107)
Level of PA (minutes per week)		
< 30	23 (28%)	10 (9.3%)
30–89	20 (24.4%)	18 (16.8%)
90–149	23 (28%)	24 (22.4%)
150–299	8 (9.8%)	39 (36.4%)
> 300	8 (9.8%)	12 (11.2%)
NA		4 (3.7%)
Do you want to be more physically active?		
Yes	62 (75.6%)	
No	17 (20.7%)	
I do not know	3 (3.7%)	
How is your physical capacity?		
Bad	18 (22.0%)	
Not so good	19 (23.2%)	
Neither bad nor good	29 (35.4%)	
Good	14 (17.1%)	
Very good	2 (2.4%)	

*Note:* Data are presented as *n* (%).

Abbreviations: CKD, chronic kidney disease; PA, physical activity.

### 3.3 Other Analysis

Table 4 outlines the association between the participants' status (person with CKD vs. nurse) and the outcome of being physically active at a moderate level minimum of 150 min/week. Being a nurse was associated with higher odds of reporting being physically active after adjustment for age and sex.

**Table 4:** Multiple logistic regression analyses of the association between the participants' characteristics and the outcome being physically active at a moderate level minimum 150 min/week.

Variables	OR [95% CI] physically active	*p*
Occupation (ref: patient)	4.68 [1.79; 12.2]	0.013
Age (years)	1.00 [0.97; 1.02]	0.826
Sex (ref: woman)	1.69 [0.53; 5.43]	0.594

*Note:* Data are presented as OR [CI 95%].

Abbreviations: CI, confidence interval; OR, odds ratio.

## 4 Discussion

This study highlights the differences and similarities between persons with CKD and nephrology nurses regarding their needs for information about PA. Persons with CKD and nephrology nurses desired more information about exercise training provided as audiovisual material, and this desire was more pronounced in nurses. Persons with CKD most frequently identified PA level as the preferred parameter for clinical monitoring, whereas nurses most often emphasized physical function as the relevant parameter. More knowledge about how to motivate patients to exercise was desired by the majority of nurses, and the majority of persons with CKD wished to be more physically active.

In the present Danish sample of nephrology nurses, the most pronounced requests were material about exercise modalities and how to motivate patients to exercise. As persons with CKD also requested more material about the effects of PA and information about different exercise modalities, there could be similarities in the materials developed to patients and nurses. By using the same material, the nurses and persons with CKD could receive the same information, and therefore their knowledge aligned to further discussion and shared understanding in clinical practice. However, when the answers from the participants in the two groups in the present study were compared, it should be noted that the groups differed in age and sex distributions. The differences between the two groups' needs were not adjusted for age and sex and may thus be confounded, whereas the data for PA were adjusted in the statistical analysis.

In a previous study, when nephrologists and dialysis nurses from dialysis units were asked if they believed that most dialysis patients would increase their level of PA if advised doing so, only 13 out of 24 dialysis nurses and 1 out of 5 nephrologists agreed (Fiaccadori et al. 2014). Therefore, it is speculated that some nurses deprioritize information about PA, if they are missing tools to motivate patients to become more physically active. This creates a discussion about the ability and accessibility of exercise and information about PA is more about personal attitudes and beliefs than equal access, tools, and knowledge. Although these findings raise some concerns, research examining the role of motivation in PA may provide valuable insights for CKD management.

Written and audio‐visual material about exercise training were more frequently requested by nurses than persons with CKD. Audio‐visual material was most frequently requested by the nurses, and this may be a result of wishing to have more nontraditional supporting material to support their patients to increase PA levels. An advantage of audio‐visual material is that it can be accessed on a smartphone, tablet, or computer within a wide range of settings. Other advantages are that videos can be used by persons with reduced literacy skills, be adapted for persons who speak different languages. They can be used together with relatives, so that they may also participate in training sessions or provide support. Other studies have looked at the emerging new tools of health information technology and agree with the finding that there is a space for new tools, particularly those which support intradialytic exercise training (Martins et al. 2025).

If new strategies to increase patients' PA levels are implemented, it may be monitored and evaluated using an outcome measure control parameter. The most obvious parameter may be a PA measure. However, a control parameter could also be physical function as this is a patient‐centred outcome that PA has positive effects on. Measuring PA may also monitor greater changes in physical function, as the two variables are correlated (Segura‐Ortí et al. 2018). In addition, while physical function is important in active daily living, the measure of PA can also be used to monitor changes related to metabolic health. While nurses reported physical function most frequently as a parameter, the persons with CKD reported PA most often. Thus, both parameters may play a key role. However, as 75.6% of the persons with CKD wished to increase the level of PA, the PA parameter may function as the most appropriate measure to support them. Persons with CKD were significantly more inactive when compared with the nephrology nurses. The difference between the two groups' PA level was not influenced by age or sex. Persons with CKD being more inactive are consistent with findings from other studies (Stevens et al. 2023). Other tests to monitor the patients' physical status in clinical practice could be physical capacity tests. The objective test of hand grip strength has been suggested, and it is easily a quickly performed (Segura‐Ortí et al. 2025). However, while the hand grip strength test is a good test to monitor overall health condition, including nutritional status, it may not be very sensitive to capture changes with exercises for the lower limbs.

It is important to mention that this study was performed in one country only and other results may be collected in other countries with other characteristics of persons with CKD and nurses. In addition, another bias was that the persons with CKD and the nurses were recruited through different organizations, and a recruitment from, for example, same nephrology centres might have reduced that bias.

This study focused on nurses as the health‐care profession that may have the most comprehensive contact to persons with CKD in clinical practice. However, other professions including medical doctors, dieticians, and physiotherapists are important to include in the work with elevation of PA levels in CKD. Thus, whilst all professions should work for the same goal of more PA with a positive attitude, every profession should do it within their knowledge and expertise. Indeed, physiotherapists have potential to play an important role in the attempt to elevate the PA level, and this could be with direct contact with the patients but also with support to the other health‐care professions with teaching of information material. However, physiotherapists may not be a prioritized staff group in all nephrology centres and thus limit the ability to collaborate (Clyne et al. 2025).

## 5 Strengths and Limitations

Despite the strengths of this study, the findings should be interpreted as exploratory and hypothesis‐generating rather than confirmatory. Whilst data were collected through national organizations, the number of participants was relatively low, and this issue might have impaired the validity of the results. The participants replied through a QR reading code, and the results may be affected by recruitment of only few participants with low technological skills, and thus, on an even more selected sample of persons with CKD. The nurses in the study may also represent a selected sample that finds research and exercise training interesting. The PA data were self‐reported and may thus be affected by recall or a measurement bias that could have been avoided if an electronic PA tracker was used. However, whilst trackers are recognized as the preferable way of measuring PA levels, questionnaires may at least in persons undergoing HD be an acceptable alternative (Alvarenga et al. 2025). Finally, we developed a bespoke questionnaire which was not fully assessed for validity and reliability, and this may have affected the results.

Furthermore, this national study did not investigate preferences for specific interventions and a programme to increase PA in persons with CKD. With respect to diverse cultures, competences, and priorities, the individual departments may develop and choose their own approach to working with PA to support the patients. Interventions could be supervised for exercise training or instructions in self‐managed exercise. However, as a minimum the staff should have regular communications about PA with their patients.

## 6 Implications for Clinical Practice

The findings suggest that PA should be addressed as part of routine conversations with persons with CKD. Both persons with CKD and nephrology nurses expressed a need for additional information about PA, including exercise modalities, safety and its potential effects. Tailored written and video‐based materials may support these conversations and facilitate shared understanding between patients and healthcare professionals. Assessment of PA and physical function may also be considered when discussing and monitoring PA in clinical practice. Although nurses have frequent contact with persons with CKD, multidisciplinary collaboration, particularly with physiotherapists, may strengthen individualised support for PA.

## 7 Conclusion

In conclusion, this study highlights a clear need for accessible information on PA and exercise training for both persons with CKD and nephrology nurses. Video‐based resources were suggested as a promising preference. PA levels were suggested as control parameters in future implementation of strategies and interventions to increase PA in persons with CKD. The findings suggest that regular dialogue about PA in clinical practice, supported by tailored written and video materials, may be a useful approach to supporting PA among persons with CKD.

## Author Contributions

**Marie Skovgaard Jakobsen:** participated in design and coordination, data analysis and interpretation, draughted manuscript, read and approved the final manuscript. **Jeanette Finderup:** participated in design of study, read and approved the final manuscript. **Hannah ML Young:** participated in design of study, read and approved the final manuscript. **Mathias Pedersen:** contributed to the setup of the database, data collection, approved and revised the manuscript. **Stig Molsted:** principal project leader, participated in design and coordination, data analysis and interpretation, helped to draft the manuscript, read and approved the final manuscript.

## Funding

The authors have nothing to report.

## Conflicts of Interest

The authors declare no conflicts of interest.

## Data Availability

The data that support the findings of this study are available on request from the corresponding author. The data are not publicly available due to privacy or ethical restrictions.
